# High initiation and long duration of breastfeeding despite absence of early skin-to-skin contact in Karen refugees on the Thai-Myanmar border: a mixed methods study

**DOI:** 10.1186/1746-4358-7-19

**Published:** 2012-12-13

**Authors:** Adrienne L White, Verena I Carrara, Moo Kho Paw, Malika, ColleyPaw Dahbu, Mechthild M Gross, Wolfgang Stuetz, Francois H Nosten, Rose McGready

**Affiliations:** 1 Shoklo Malaria Research Unit, PO Box 46, Mae Sot, Tak, 63110, Thailand; 2 Midwifery Research and Education Unit, Department of Obstetrics, Gynaecology & Reproductive Medicine, Hannover Medical School, Hannover, Germany; 3 Institute of Nutrition, Friedrich-Schiller-University, Jena, Germany; 4 Centre for Tropical Medicine, Nuffield Department of Clinical Medicine, University of Oxford, Centre for Clinical Vaccinology and Tropical Medicine, Oxford, UK; 5 Mahidol Oxford Research Unit, Faculty of Tropical Medicine, Mahidol University, Bangkok, Thailand

**Keywords:** Breastfeeding initiation, Breastfeeding duration, Early skin-to-skin care, Midwifery, Refugee, Resource-poor, Swaddling

## Abstract

**Background:**

Early skin-to-skin contact (SSC) after birth is recommended as part of the United Nations Children’s Fund (UNICEF) baby friendly health initiative to promote optimum breastfeeding. This paper reports rates of breastfeeding initiation and duration in a low resource environment, where early SSC is not practised, and explores views of pregnant women and midwives surrounding breastfeeding and swaddling.

**Methods:**

Data from records from a single hospital on the Thai-Myanmar border where refugee women gave birth during a one-year period (2010) were used to determine breastfeeding initiation rates and the time of the first breastfeed, and duration of breastfeeding of the previous alive child in multigravidae. Focus group discussions (FGD) were conducted to obtain information from pregnant women attending antenatal care about their intended or previous duration of breastfeeding and views on breastfeeding. Interviews with local midwives explored reasons for high rates of breastfeeding in this setting and the practice of newborn swaddling.

**Results:**

Of 1404 live births in 2010 in Maela refugee camp there were 982 evaluable mother-newborn pairs, including 80 infants born before 37 weeks gestation. Initiation of breastfeeding within the first hour after birth and exclusive breastfeeding at discharge in term mother-newborn pairs was 91.2% (823/902) and 99.3% (896/902); and before 37 weeks gestation, 48.8% (39/80) and 98.8% (79/80). Reported duration of previous breastfeeding was 19 (range 2 to 72) months.

During FGD all primigravidae (n = 17) intended to breastfeed and all multigravidae (n = 33) had previously breastfed; expected or previous duration of feeding was for more than one year or longer. The major theme identified during FGD was breastfeeding is “good”. Women stated their intention to breastfeed with certainty. This certainty was echoed during the interviews with midwifery staff. SSC requires a delay in early swaddling that in Karen people, with animistic beliefs, could risk loss of the spirit of the newborn or attract malevolent spirits.

**Conclusions:**

In a population with a strong culture of breastfeeding and robust breastfeeding practices, high rates of initiation and duration of breastfeeding were found despite a lack of early skin-to-skin contact. Local preferences, traditions and practices that protect, support and maintain high rates of breastfeeding should be promoted.

## Background

Early skin-to-skin contact (SSC) by placement of the naked baby, head covered with a dry cap and a warm blanket across the back, prone on the mother’s bare chest at birth is described as the physiological norm and is recommended globally as standard practice and as part of UNICEF baby friendly health initiative [1]. Introduction of SSC into standard care of healthy newborns has been shown to confer both immediate and long-term beneficial outcomes for mother and baby [1]. A recent Cochrane review to assess the effects of SSC on breastfeeding (and other parameters) has analysed 34 randomized controlled trials with 2177 mother and baby pairs, including rates of: breastfeeding at 1–4 months, exclusive feeding at 3–6 months and duration of breastfeeding [2]. The authors report that SSC shows statistically significant positive effects on breastfeeding with the caveat of cautious interpretation because of small samples, varied contexts and heterogeneity of study outcomes. They recommend that SSC be discussed with parents prior to birth and incorporated into routine newborn care. A systematic review of interventions that promote or inhibit breastfeeding in neonatal units found that any SSC significantly increased breastfeeding duration, as did peer support in hospital and family support at home [3]. Again, this was presented with a caveat: cautious interpretation as other interventions such as staff training in breastfeeding support may interrelate and SSC may not be as effective if used alone. Unsupervised SSC was recently raised as a concern in a series of apparent life threatening events in presumably healthy term newborns during early SSC in unattended mothers [4]. We identified two SSC randomized controlled trials published since the Cochrane review and both assessed feeding behaviour [5,6]. No significant impact of SSC was observed in New Delhi, India [6] with 41 mother-infant pairs using the modified infant Breast-feeding Assessment Tool [7], while a significant difference was reported in Islamabad, Pakistan [5] in 183 mother-infant pairs, using the same tool. In both sites, exclusive breastfeeding rates (at one month [5], and at 6 weeks [6]) were significantly better in the SSC group.

Two randomized controlled trials with a special consideration of swaddling were identified. SSC and early suckling, or both, had a positive influence on mother-infant interactions at one year as assessed by Parent–child Early Relational Assessment [8]. Suckling initiated within two hours of birth where the mother and baby were close together but not skin-to-skin compensated for the lack of SSC in swaddled babies [9]. In the other trial swaddling alone did not influence the number of breastfeeds, the duration of each feed or the volume of milk ingested but combined with separation of mother and baby had deleterious effects on both breastfeeding and neonatal weight gain [10]. These studies suggest that some of the negative effects of swaddling may be ameliorated by early and exclusive breastfeeding and avoidance of mother-infant separation after birth.

Breastfeeding rates are influenced by culture, including social and environmental factors, mothers’ understanding of the consequences of not breastfeeding, and family and community support for breastfeeding [11,12]. Extremely poor breastfeeding and complementary feeding practices in developing countries have previously been unrecognized [13] and much less is known about these practices in refugee populations.

Using a mixed methods study design we aimed to document breastfeeding initiation and duration rates in Karen women from Myanmar living in Maela refugee camp on the western border of Thailand. We also describe the views of pregnant women and midwives about breastfeeding and the views of midwives on universal early swaddling in a setting where early SSC is not practiced.

## Methods

### Setting

**Figure 1:**
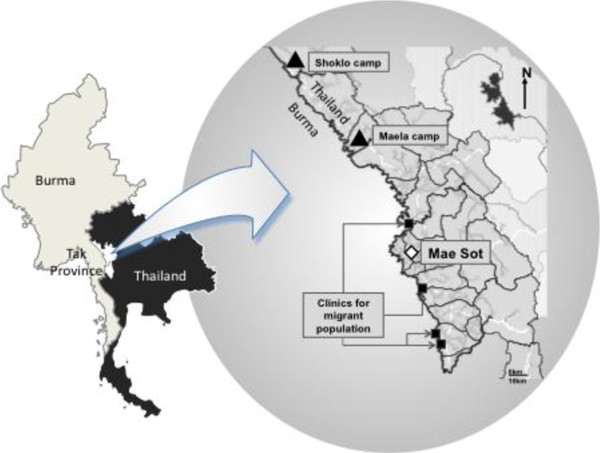
Map of the study area.

There are an estimated 160,000 refugees from Myanmar living in camps along the western border with Thailand. Maela is the largest of the refugee camps with a population of 47,955 refugees situated on the Thai side of the border [14]. Most of the refugees on this part of the border identify as ethnic Karen from Myanmar, speak up to five different languages and dialects and have Buddhist, Christian, Muslim and animist religious beliefs. The Shoklo Malaria Research Unit (SMRU), based in Mae Sot, has been doing operational field-based research for the past 26 years. Since its inauguration SMRU has also provided maternity services to refugees, formerly in Shoklo (until 1995 when Shoklo was moved to Maela) and currently in Maela camp (Figure 1). Approximately 1500 women per annum give birth at SMRU facilities in Maela refugee camp.

Provision of education and other medical services in Maela camp is facilitated by various non-government organisations. The main health provider in Maela camp is the non-government organization Aide Médicale Internationale. In 2010 limited food rations of rice and beans, supplemented by oil and tinned fish were provided to pregnant women [15]. Fresh vegetables are sometimes grown in or around the camp, but space is scarce so most fresh food is transported in from outside the camp.

Refugees live in densely spaced bamboo and thatch houses with basic sanitation. Water is available from communal wells, and limited electricity is available for essential services. Forty nine percent (49%) of the population are aged less than 17 years of age and can attend primary and secondary school in the camp. Opportunities for higher education or employment within the camp are extremely limited [16]. Camp services include basic medical care, community health clinics and places of worship [14].

### SMRU maternity unit

SMRU maternity unit provides full antenatal care, including ultrasound gestational age assessment by local health workers (since 2001) [17], care for all women having vaginal births, (including breech and vacuum birth) and postnatal care of mother and baby. All pregnant women attending SMRU antenatal care are provided with folate, iron, and thiamine supplements, tetanus vaccination, health information and advice about diet in pregnancy and during lactation. HIV testing for prevention of mother to child transmission was introduced in the refugee population in 2001.

Traditionally women give birth at home and SMRU has always encouraged women who attend antenatal care at SMRU to present to the birth unit in labour. For the past 10 years approximately 25% of women have continued to birth at home. Women needing caesarean delivery are referred to Mae Sot hospital in Thailand, an hour away by car. SMRU is a midwife-led unit with clinical and 24-hour telephone back up from local and expatriate medical doctors and the World Health Organisation partograph has been used since 1995. Recently, there has been implementation of a formal skilled birth attendant (midwifery) curriculum. In this manuscript the term midwife is used to describe skilled birth attendants. The unit has developed evidenced-based clinical protocols for locally trained medics, midwives and nurses.

SMRU has maintained a system of childbirth records for all women who attend antenatal care and these have been entered to a computerised database since 1986. The record is a similar design to a care plan and is routinely used by midwifery and medical staff. One of the antenatal enrolment questions includes duration of breastfeeding of the last child. One of the standardized charts is the postpartum observation of mother and newborn for infants born in the clinic (Additional file 1). This chart specifies the time of the first breastfeed and every breastfeed in the first 4 hours. As an operational field based research unit SMRU has a vested interest in ensuring the charts and records are filled in correctly and are suitable for digital data entry. Birth records are double checked by senior medical staff before data entry. A WHO safe motherhood needs assessment in Maela camp has found very high accuracy of maternity record keeping [18].

### Care in labour

**Figure 2:**
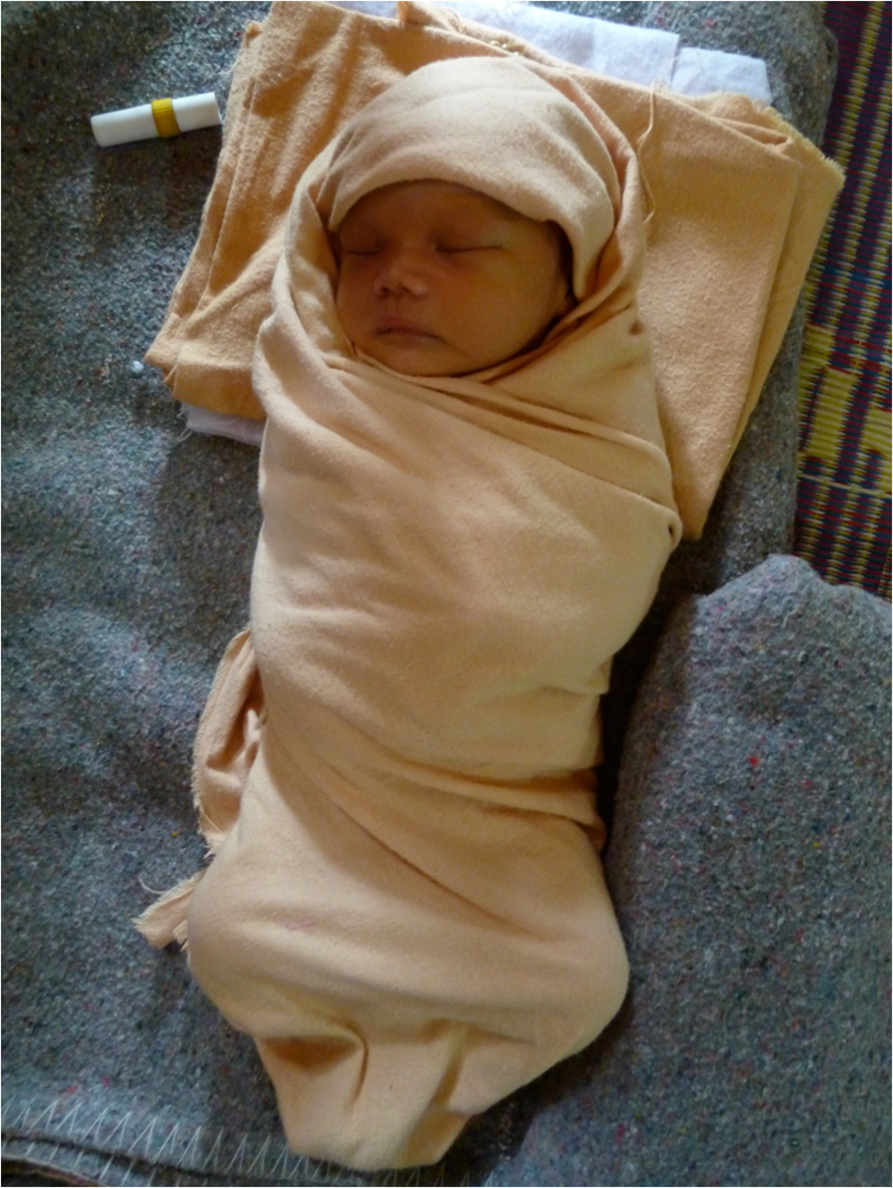
Swaddled Karen newborn.

During normal labour at SMRU maternity unit, each woman is cared for by a locally trained midwife and is usually also accompanied by a female relative, her traditional birth attendant, the baby’s father or a neighbour. She is strongly encouraged to eat and drink, remain upright and mobile, returning to the labour room for regular observations. Management of pain includes massage, local application of heat, showering and walking but narcotic and other forms of pain medication are not available in this setting. Women do not usually vocalize their pain during labour. During the second stage of labour the woman is attended continuously by her midwife and encouraged to remain upright, maintain fluid intake and pass urine frequently. After the cord is cut the baby is dried and placed on a warmed surface for immediate newborn care and then wrapped in one or two large thin cotton swaddling cloths that also cover the head (Figure 2). Swaddling of Karen babies differs from that described in other literature pertaining to swaddling and SSC [9,10]. SSC is not currently practiced in this population. The mother and baby period of separation is brief (usually 15–20 minutes) and mother and baby remain in the same room together.

### Postnatal care

**Figure 3:**
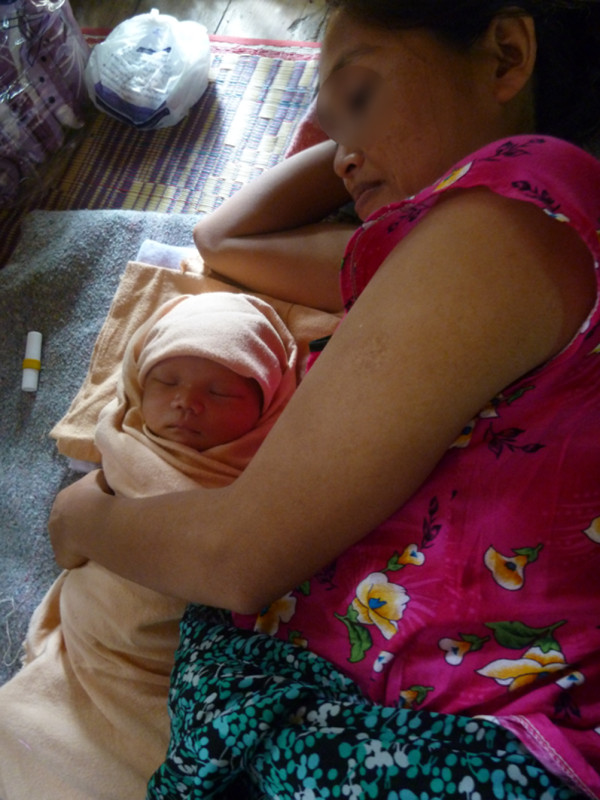
Rooming-in Karen mother and baby.

**Figure 4:**
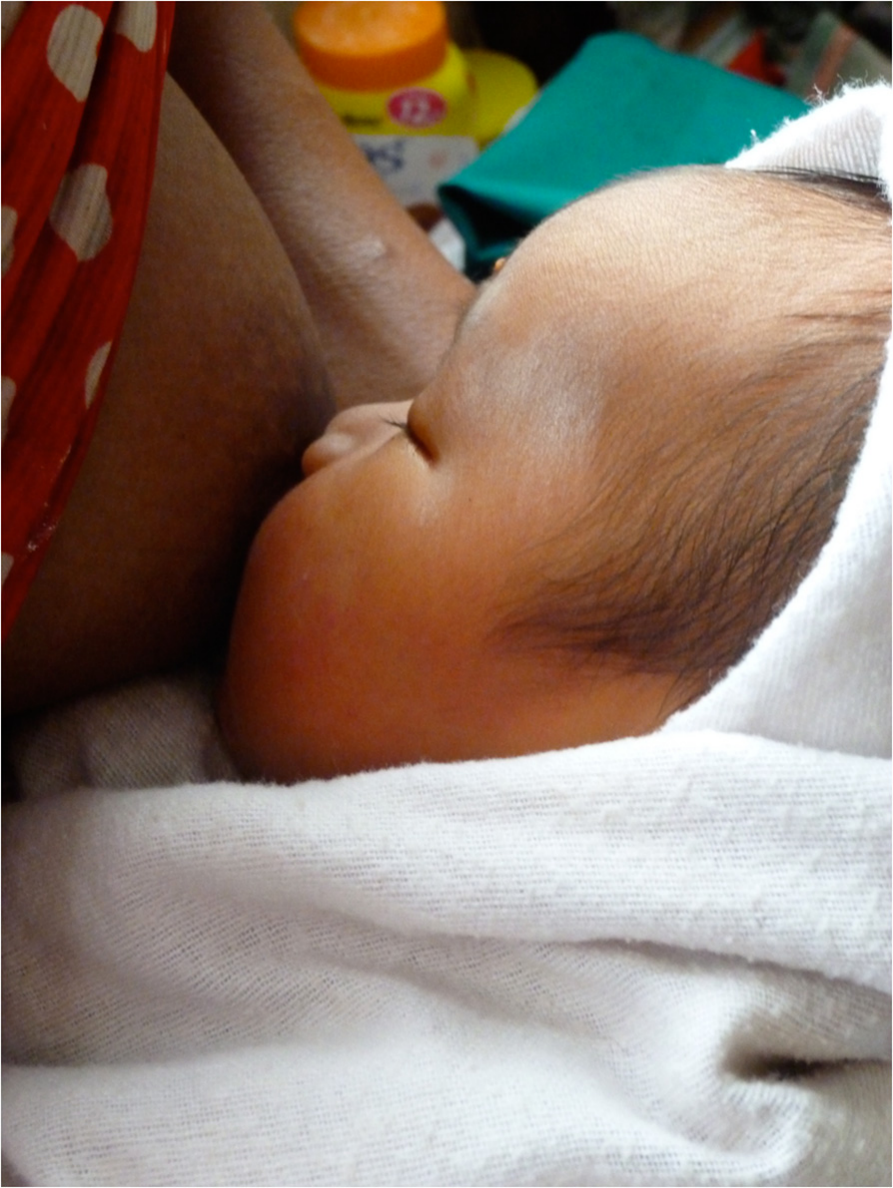
Breastfeeding a swaddled newborn.

Rooming-in is universal and neither cots nor beds are traditionally used in this culture. In the home, families sleep together on woven mats on the bamboo floor (Figure 3). Babies remain swaddled during feeds and the SSC is minimal (Figure 4). Newborn swaddling cloths are only removed during bathing, changing of soiled cloths or for medical examinations. Midwives encourage early and frequent breastfeeding and women often have support from older female relatives or neighbours who have breastfeeding experience. Frequent breastfeeding (8–16 times per day) in response to baby cues is common and teats or pacifiers (dummies) are either unknown or rarely used. Babies are carried in their mother’s arms, in a sling or laid on the mat when mother is busy, usually under a net to protect from mosquitoes. Crying babies are picked up immediately. Fathers take an active role in caring for the mother and baby postnatally. Most women go home 24-hours after birth and are encouraged to return if they experience problems. Mothers return at one month for newborn checks and routine postnatal follow-up is provided at the clinic run by Aide Médicale Internationale.

### Special care baby unit

Special newborn care is provided in a unit where mothers and babies share the same design of sleeping place as mentioned above. Incubators are rudimentary and in short supply, so are prioritised for phototherapy and newborns with very low birth weight. If milk supplements are needed, fresh donor breast milk is obtained after screening for HIV and hepatitis B. Infant formula is used in preterm infants only when donor milk is unavailable. Provision of powdered infant formula and the equipment and education needed to safely prepare feeds is otherwise reserved for adoption, severe cleft lip/palate, maternal death and untreated HIV.

When infants are transferred to special care baby unit the standardized postpartum observation chart for the newborn (Additional file 1) is discontinued.

### Extraction of data from birth records

Data from birth records with a date of birth from 1 January 2010 to 31 December 2010 were extracted if the infant was born alive, with a normal examination, a gestation of 32 weeks or more, (as this is the gestation when the suck/swallow reflex is usually functional [19]), and did not die in the perinatal period. Only specified data was extracted and included: maternal age, parity, breastfeeding history (of the last live-born child), gestation at birth, and whether the baby was breastfed within the first one hour of birth. Two midwives independently extracted the data, entered and checked it for discrepancies by verification with the original record. The cumulative rate of breastfeeding was available for each hour of the first 4 hours. When the baby was not breastfed within four hours of birth the midwifery notes were searched to find the time of the first breastfeed. Special care baby unit records were searched in the event of transfer. Detailed recording of breastfeeding initiation was unavailable for women transferred to the local Thai hospital for caesarean birth and those who chose to birth at home; therefore these births were excluded from this analysis.

### Focus group discussions

During the last week of April 2011, focus group discussions (FGD) were conducted using a purposive sample of currently pregnant women at antenatal clinics, as they would be likely to be willing to talk about their breastfeeding experiences and intentions. Nine focus groups were conducted from Monday to Friday with four to eight women per group (space limited to eight), depending on the numbers present at the antenatal clinics. Women attend antenatal clinic on the day of the week specified for their section of the camp. Women were asked if they wished to participate by the midwife in charge of antenatal clinics, who was not involved in other aspects of the study.

To avoid confounding, FGD of primigravidae and multigravidae were held separately. In this setting, older people and those in authority are accorded great respect, so combining women in their first pregnancy with older, more experienced mothers could potentially inhibit the younger women [20]. Three groups of primigravidae and six groups of multigravidae were planned in accordance with the general proportions of these in this population (ratio of 1:2).

Use of a questionnaire was avoided as literacy rates are less than 50% in pregnant women in this population [21], and focus groups were able to include those unable to read or write. One midwife fluent in the local languages and in English, with experience in group counselling and focus group facilitation [22] co-facilitated, translated and interpreted at every FGD. The questions in this FGD were limited and the co-facilitator piloted the questions in the week before the FGD were conducted. The participants were seated in a circle on the bamboo floor with the researcher and the translator. To allow attention to focus on the co-facilitator the researcher sat a little outside the circle. The researcher was known to the participants but not involved in their clinical care. Each group session was held in the health education room, a secluded area adjacent to the antenatal clinic and lasted 20–30 minutes. Drinks and snacks were provided. Responses during each FGD were translated into English and recorded in note form by the researcher and later transcribed. All names have been changed to pseudonyms to ensure confidentiality.

During the FGD simple information about women’s intentions, or experience of duration of breastfeeding was used to stimulate discussion with the aim of ascertaining more nuanced information to add to the quantitative data. Questions included three open-ended questions and two, fixed-response questions, but the groups were encouraged to discuss any points that were raised and spontaneous conversations were not interrupted [22]. Women were reminded that they could speak at any time. The FGD started intentionally with an “ice-breaker” question to bring focus to the topic and to encourage laughter and cross conversation: *How were you fed as a baby?* First-time mothers were then asked the following closed questions: *Do you plan to breastfeed? Do you plan to feed for more than 3 years, more than 2 years, more than 1 year, more than 6 months?* (Women were asked to raise their hands in response [22]). Other open-ended questions were: *Can you tell me about breastfeeding? How do you know about breastfeeding?* Multiparous women were asked the same set of questions however the question pertaining to duration was about their last baby: *Did you feed your last baby for more than 3 years, more than 2 years, more than 1 year, more than 6 months?* (Again, women were asked to raise their hands in response).

### Interviews with experienced local midwives

Experienced local midwives involved in a train the trainer program in Maela refugee camp were approached to participate in semi-structured interview by the teacher of the program (also a researcher [ALW]). Three of the six midwives declined, each stating her reason as not being a mother. The participating midwives each had more than 15 years' experience and were fluent in English and had themselves given birth in the refugee camps or Thai hospitals. None were involved in the FGD. Interviews took place over two days in May 2011 and lasted between 30–45 minutes. The interviews were conducted in a private area near the clinic at a time of the midwives choosing.

"They were asked the following open-ended questions:"

"Can you tell me about breastfeeding in your [Karen] culture? Can you tell me how women know about breastfeeding? When we ask pregnant women at FGD about breastfeeding they all want to do it – why do you think this is so?"

Universal swaddling is observed to occur at every birth. To understand swaddling in this population semi-structured interviews were scheduled for September 2012 with the same midwives. The questions were:

"I see that babies are swaddled immediately after birth, in the postpartum unit, during breastfeeding and whenever their mothers bring them back to the clinic. Can you tell me about swaddling and why women always swaddle their babies?"

These interviews in English were recorded by the researcher and later transcribed. The midwives were asked to read the transcribed document to verify if the meaning remained the same.

### Ethics /informed consent

For the extraction of data, ethical approval for retrospective analysis of pregnancy records was given by the Oxford Tropical Research Ethics Committee (OXTREC 28–09, amended 19 April-2012). Attending antenatal care was voluntary as was women’s participation in FGD. Verbal consent was obtained for FGD as no risk was involved for the pregnant woman. Women were assured that all data would remain confidential. Midwife consent was obtained in writing and participation was voluntary. All responses have been de-identified and names have been changed to ensure confidentiality.

### Data analysis

Breastfeeding initiation and duration, parity and maternal age data was entered into an Excel (2007) spreadsheet. Proportions and the Chi-squared test for linear trend was analysed in SPSS for Windows version 14·0 (SPSS, Inc., Chicago, Illinois, USA).

Two researchers, one with more than 18 years experience in this population, independently read and re-read transcripts of FGD and interviews. The transcriptions were sorted into meaning units [23]. Three major themes emerged in relation to breastfeeding and two in relation to swaddling due to this procedure. Any disagreements were resolved through discussion.

## Results

**Figure 5:**
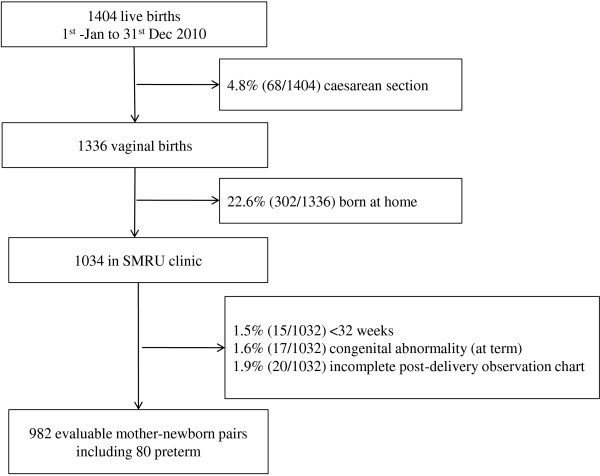
Evaluable mother-newborn pairs in 2010.

Of 1404 live births in 2010 in Maela Refugee camp there were 982 evaluable mother-newborn pairs (Figure 5). Of the 982 pairs, 1.3% (13/982) were twin births and seven of these were at term. There were 8.1% (80/982) of births with a gestational age between 32+0 and 36+6 weeks. There were 35.9% (353/982) primigravidae and 64.1% (629/982) multigravidae, with a mean [min-max] age of 21 [14–42] and 29 [15–47] years. The median parity of multigravidae was 2 [1-10].

### Breastfeeding initiation rates

**Figure 6:**
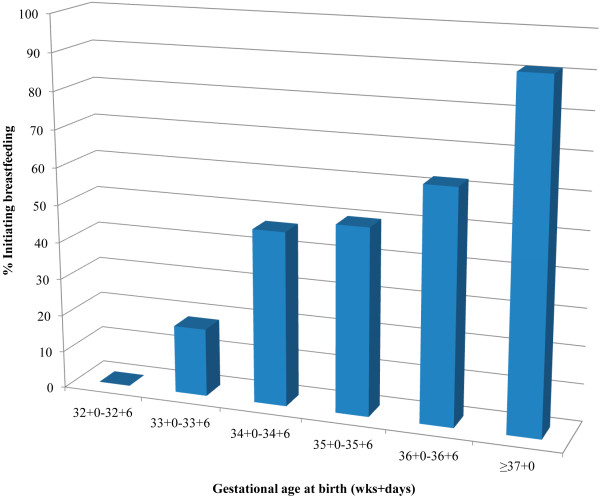
Proportion mother-newborn refugee pairs initiating breastfeeding in the first hour according to gestational age in 2010.

**Figure 7:**
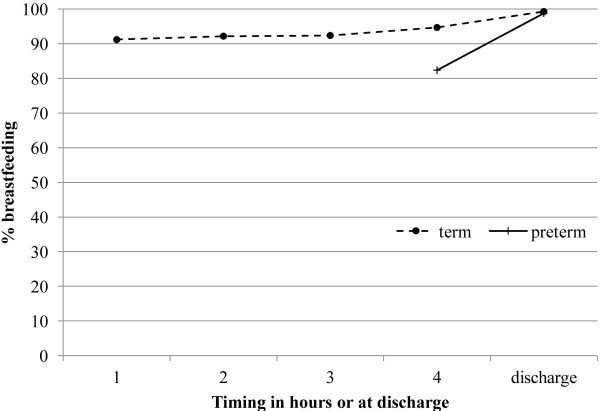
The cumulative proportion of term and pre-term mother-newborn refugee pairs initiating breastfeeding within four hours of birth and by discharge in 2010.

Initiation of breastfeeding within the first hour after birth in term mother-newborn pairs was high, 91.2% (823/902) (Figure 6). The cumulative proportions of mother-newborn pairs initiating breastfeeding within two, three, and four hours after birth was: 92.2% (832), 92.4% (834) and 94.7% (855), respectively (Figure 7). At discharge from hospital 99.3% (896) were exclusively breastfed including 31 mother-newborn pairs admitted to special care nursery. The six mother-newborn pairs who did not initiate feeding included two neonatal deaths. The other four received powdered infant formula as three of the mothers had HIV and one baby was adopted.

In mother-newborn pairs with a gestational age at birth between 32+0 and 36+6 weeks, initiation of breastfeeding within the first hour after birth was 48.8% (39/80). The proportion of breastfeeding in the first hour after birth increased significantly with increasing gestational age: 0% (0 of 3), 18.2% (2/11), 46.7% (7/15), 50.0% (7/14), 62.2% (23/37) at 32, 33, 34, 35 and 36 weeks, respectively (P = 0.003, linear trend) (Figure 6). In the remaining 51.2% (41/80) mother-newborn pairs where initiation did not occur in the first hour, exact breastfeeding initiation time was identified in 70.7% (29/41) of records. Initiation of breastfeeding occurred within 4 hours in 41.5% (17/29) and by 24 hours in the remaining 58.5% (12/29) pairs. At the time of discharge 98.8% (79/80) of these preterm mother-newborn pairs were exclusively breastfeeding. One pair was discharged supplementing breastfeeding with infant formula in twins born at 35+5 week’s gestation.

### Previous breastfeeding duration

The median duration of previous breastfeeding was 19 (range 2 to 72) months in the 90.3% (568/629) of multigravidae, who had a live-born, congenitally normal infant that was not a neonatal death.

### Focus group discussion

A total of 50 women, including 17 primigravidae and 33 multigravidae participated in the nine FGDs. When asked the icebreaker question about how they were fed as babies, there was often laughter and surprise that the question was serious. The opening question encouraged women to speak to each other and they expressed amusement at the notion that they may not have been breastfed. Non-verbal communication was observed during FGD including nodding in agreement with another’s opinion or experience.

When asked, 100% (50/50) of women expressed their intention to breastfeed or their experience of breastfeeding with certainty. In primigravidae the intended and in multigravidae the actual duration of breastfeeding was more than one year for 96% (48/50). One multigravida breastfed her last child for only three months as she went to work outside the camp and only one primigravida showed any hesitation in her intended length of breastfeeding; “*If the baby is healthy I will stop early; after 6 months, or [I will breastfeed] until I am pregnant again; if the baby is not healthy I am not sure.”* (Paw Mue, first pregnancy, aged 20 years). Another women in her group spontaneously assured her that she could breastfeed for longer if she wished.

Three major themes emerged from the qualitative data: 1) Breastfeeding is “good”, 2) Bottle feeding is for other people and 3) My mother told me about breastfeeding.

#### Breastfeeding is “good”

The strongest theme to emerge was from positive statements about breastfeeding and the acknowledgement of breastfeeding as an inherent part of being a mother:

"“I breastfed for more than three years because I have enough milk [laughing]” (Moo Thu Awah, mother of two, aged 29 years). The expression ‘enough milk’ in this community is used to mean plenty of milk, so this woman continued to feed because she had an abundant milk supply. Almost all women who spoke during the FGD talked about breastfeeding as something they did for the benefit of the baby:"

"“[Breastfeeding] will make my baby strong, have [a] good brain” (Mu Mu, first pregnancy, aged 26 years, plans to breastfeed for more than 2 years). Other women used phrases or words with a similar meaning such as ‘good’, ‘strong’, ‘healthy’, ‘smart’, and ‘protection against diarrhoea’ or ‘protection against infection’."

A longer duration of breastfeeding was expressed as an expected commitment to the wellbeing of the baby:

"“I feel pity for the baby if breastfeeding [is] stopped before one year as the baby is too small” (Khee Lar Say, first pregnancy, aged 23 years, plans to breastfeed for more than 2 years) and:"

"“I will breastfeed for more than one year- until my baby is big enough for other food, after 14 months my baby is big enough for other food” (Mu Chai, first pregnancy, aged 21 years, plans to breastfeed for more than one year),"

"“I breastfed for one year, seven months, the baby stopped because she didn’t like [to breastfeed] and [wanted] to eat other food.” (Lwe Lahr Paw, mother of one, aged 26 years, breastfed her baby for more than one year), or a similar commitment to the health of the baby:"

"“This is my only [first] baby so I want the baby to be very healthy” (Ma Kyi Aye, first pregnancy, aged 20 years, plans to breastfeed for more than 3 years)."

Some women also gave examples of how breastfeeding would affect both them and their baby:

"“If I breastfeed it is good for the baby, protect for infection, if I give other wrong food the baby will get sick. [She paused and then added] my uterus will get smaller.” (I Shar, mother of one, aged 25 years, breastfed for more than 2 years)."

Women sometimes gave responses that reflected their positive view of breastfeeding by stating what would happen if they did not breastfeed:

"“If I don’t breastfeed, my breasts will become very big and hard.” (Naw Say Khu, mother of two, aged 27 years, breastfed for more than 2 years). [She later added] “If I breastfeed [my daughter] she will grow up to breastfeed.”"

#### Bottle feeding is for other people

Within all except one of the focus groups the topic of bottle feeding was spontaneously introduced. Unlike breastfeeding, bottle feeding was not discussed as a personal experience but as something other people did. The statements about bottle feeding were contrary to those about breastfeeding:

"“[I saw] the baby bottle feeding and [the] baby gets sick; bottle feeding is not good because of diarrhoea” (Sha He Dar, first pregnancy, aged 24 years, plans to breastfeed for more than 2 years) and:"

"“Other people have bottle feeding if they have HIV, [If they bottle feed] then people will think they have HIV.” (Naw Mu, mother of two, aged 31 years, plans to breastfeed for more than one year),"

"“Breastfeeding is easy, if my baby cries I can feed him straight away, bottle feeding takes a long time, bottle feeding is difficult” (Phu Maung, mother of one, aged 25 years, breastfed for more than 2 years)."

#### My mother told me about breastfeeding

When asked how they knew about breastfeeding, almost all women acknowledged their mothers as a primary source but did not elaborate on this, just made statements such as:

"“My mother told me about breastfeeding.” (Moo Yay, mother of one, aged 24 years, plans to breastfeed for more than one year)."

"In each group, once one woman volunteered this information during a FGD, others mostly just nodded in agreement."

Women also talked about community sources of breastfeeding information. These included; the Karen Women’s Organisation (a community based organisation established in 1949), other camp based community groups and non-government organizations, camp libraries, the antenatal clinic, the medical clinic run by Aide Médicale Internationale, schools and ‘Health Messenger’ (a locally produced magazine for health-workers in Burmese and English).

### Interviews with experienced midwives

Similar themes emerged from the transcripts of midwifery interviews as FGD sessions. Midwives emphasised the role of mothers and grandmothers as the source of their understanding of breastfeeding and made little mention of community sources of information.

#### My mother told me about breastfeeding

When talking about why women breastfeed one midwife said:

"“Everybody breastfeeds because grandmother tells mother and mother tells daughter and shows them how to do it and tells them they must do this and helps them if they cannot.” (Ju May Paw, aged 47)."

The midwives, like the women, gave responses that suggested an acceptance of breastfeeding as an unremarkable and integral aspect of being a mother:

"“Every woman gives breastmilk after delivery. This is usual. Everybody must do this. [We know] breasts will be full otherwise –if she cannot [breastfeed] her mother or grandmother will help her by squeezing out [expressing milk] or show her how to do it” (Chit Su, aged 40),"

"“They know the baby will be born hungry so they give milk first [immediately after birth]” (Ju May Paw, aged 47)."

One midwife drew on her personal experiences to illustrate her views:

"“Nobody told [showed] me about breastfeeding –I just knew I would breastfeed when I had my baby. Our women are strong and we always breastfeed our babies so that they will be strong and healthy too” (Chit Su, aged 40)."

#### Bottle feeding is for other people

Again, the topic of bottle feeding was spontaneously introduced by the midwives and the idea that bottle feeding is for others also emerged as a clear theme:

"“It was difficult to find powder-milk [infant formula] in Karen state [state in Eastern Myanmar mostly populated by ethnic Karen], so everyone gives breast milk.” (Ju May Paw, aged 47),"

"“If the woman is rich she can give powder milk, but our women are not rich so we cannot buy powder milk.” (Chit Su, aged 40),"

"Some [women] will go back to work after 2 or 3 months then they might give powder-milk” (Ju May Paw, aged 47)."

### Swaddling at birth

In this community swaddling soon after birth and during the first month of life is believed to calm the newborn and prevent crying. Two major themes emerged from the data: 1) The baby will not feel afraid and 2) Protection from spirits.

#### The baby will not feel afraid

All three midwives shared views in support of swaddling to calm the baby and make them feel secure:

"“Swaddling calms the baby. If they are not calm this can lead them to start to cry.” (Mu May, aged 45),"

"“If the baby cannot move they will not feel afraid and will not have jerking of the arms. If wrapped, the baby will be warm and sleep a long time. If you want to pick her up, you know and breastfeed, it is easier [to do this] if the baby is wrapped.” (Chit Su, aged 40),"

"“Swaddling is for the baby to get the feeling of being held like you would give a proper hug to someone you care for.” (Mu May, aged 45)."

"“Swaddling means legs [and] arms [are] not curled, how do you say, not straight? If curled we believe look not beautiful”, [she paused and added, laughing], “but this is not true.” (Ju May Paw, aged 47)."

Swaddling was also believed to play an additional role in the health of newborns as it could assist the parents to recognize problems in the baby:

"“A normal child will be calm just with being hug [ged] and proper [ly] wrapped, if the child cries more than usual it indicates something wrong, which extra attention need to be taken like going to hospital or get advice.” (Mu May, aged 45)."

A second theme to emerge was about good and bad spirits and how swaddling protects the baby and the family from spirits, although not all the midwives had heard about or believed this.

#### Protection from spirits

"“We believe that the spirit[s] of the newborn baby are sensitive, [and] can be slip away from their body easily, [which is] why we must tuck [swaddle] them tightly.” (Mu May, aged 45) and: “If the baby cry all the time it is believed to be bothered by the bad spirit, the over crying of the baby may bring bad luck to the parents [and] can be refer to [the spirits’] wish for the death of their parents, which [is] one of the reasons why you don’t want baby to cry too long and try to solve the mystery as soon as you can.” (Mu May, aged 45),"

"“ If the baby is taken outside, [we swaddle] to stop the baby crying a lot; if they cry a lot the spirits will catch them. If we go out somewhere we must use ginger or galangal [aromatic roots, commonly used in cooking] to protect against bad spirits.”(Ju May Paw, aged 47). It is common to see a piece of galangal or ginger placed near the newborn’s head or pinned to their swaddling cloth."

It is interesting that not all midwives mentioned animist beliefs when discussing swaddling and even after prompting did not agree that a belief in spirits was linked to swaddling practice:

"“I don’t know [about spirits]; I will ask my aunty” [she spoke to her aunty on the phone] “My aunty does not know about spirits, maybe Buddhists know about that [laughing]” (Chit Su, aged 40)."

## Discussion

In this environment SSC is not practiced and newborns are universally swaddled; mother and infant room-in from birth until hospital discharge. In the first hour of life 92% of term and 49% of preterm infants initiated breastfeeding, with nearly 100% of all newborns initiating breastfeeding and exclusively breastfeeding at discharge. This could be explained in part by high normal vaginal delivery rates without maternal analgesia and minimal separation of mother and newborn [24]. Single strategies are unlikely to increase breastfeeding initiation as stand-alone measures. The reported median duration of previous breastfeeding was 19 (range 2 to 72) months approaching the WHO recommendations of continued breastfeeding up to 2 years [25]. Primigravidae anticipated they would breastfeed for more than one year or longer. This is similar to a review of feeding practices from nine Asian countries where Myanmar compared favourably to the other countries with overall rates of: 99.9% (95% CI: 99.8, 99.9) for any breastfeeding and 67% (95%CI: 63.2, 71.4) for continued breastfeeding at 2 years [26]. However, temporary work in Thailand that necessitates refugees moving in and out of the camp reduces the social isolation that has in the past afforded some protection against risky health behaviours [27,28]. Karen refugees are increasingly exposed to infant feeding practices within Thailand where there are much lower recommendations for exclusive breastfeeding [29]; low reported rates of breastfeeding [14,29-31]; and highly visible commercial marketing and promotion of infant formula [29]. At the time of writing, breastfeeding practices remain resilient in this refugee population with apparent strong and effective family and community supports [32].

There was an implicit acceptance by women and midwives that breastfeeding was something inherent to motherhood and “good” for their babies, similar to views expressed by women in a resource rich environment [33]. The women and midwives did not regard breastfeeding as something special, nor long duration of feeding as something unusual or remarkable. On the contrary, short duration of breastfeeding was unusual, and women were apologetic when they spoke of this. Women talked about difficulties with breastfeeding such as engorgement as a problem to be overcome rather than a reason to stop breastfeeding. A recent systematic review of factors associated with duration of breastfeeding has identified women’s breastfeeding intention, breastfeeding self-efficacy (confidence) and social support as important for high levels of initiation and duration [34]. Intention to breastfeed was a strong predictor of positive breastfeeding outcomes as was knowledge of health effects of breastfeeding, which increased both intention to breastfeed and duration of breastfeeding [35,36]. The high intentions in this population correspond to the observed high overall breastfeeding rates.

Karen women knew about breastfeeding predominantly from their mothers with knowledge passed from older female relatives to younger ones. Women gave other examples of sources of breastfeeding information within the camp but few mentioned midwives or the SMRU antenatal clinic. Women in this population reported strong views on bottle feeding that were mostly associated with illness in both mothers and babies. This is interesting given the rarity of the use of bottles, relatively high cost of infant formula and very low rates of HIV in this population (< 0.5% of the pregnant population) [28]. When SMRU started the prevention of mother to child transmission of HIV in 2001 in the refugee camp three of the first six infants died from bottle fed diarrhea. A concerted effort to ensure correct bottle feeding practice amongst these women was required. With the 2009 WHO PMTCT Guidelines SMRU rapidly adopted breastfeeding for women on triple therapy rather than advocating bottle feeding [37] given the strong culture of breastfeeding within the community.

Early SSC is not practised in this setting and babies are always swaddled as soon as possible after birth. This led us to enquire about the practice of swaddling in this community. The major themes that emerged about swaddling was that it is beneficial to the baby and protects against spirits. SSC requires a delay in early swaddling which in Karen people, with animistic beliefs, would risk loss of the spirit of the newborn or attract malevolent spirits [38]. Despite conversion to Buddhism and more recently, to Christianity, Karen animist beliefs are strongly evident in day-to-day life [38]. The possible ill effects of swaddling on breastfeeding described elsewhere [2,10] may have been ameliorated in this population by close early contact, early breastfeeding, lack of separation of mother and infant from birth and strong motivation to breastfeed.

The main limitation of this study was no data on exclusive breastfeeding at 6 months of age as one of the main benefits of early SSC is increased exclusive breastfeeding at six months [2] and subsequent reduced child mortality especially from diarrhea and pneumonia [39,40]. There was also no data available on breastfeeding rates for women transferred to hospital in Thailand for caesarean section, or who delivered at home. It is unlikely the population has been misrepresented as the majority of births were included in the analysis. Midwives who were not mothers declined to be interviewed which could potentially have led to a bias in views of breastfeeding and swaddling. It was not surprising these midwives declined to be interviewed given the culture of respect and deferral to age and experience. In-depth interviews with mothers and midwives may have revealed more complex information about breastfeeding in this culture. Alternatively there may not be more complex reasons in an environment where bottle feeding is not a viable option and where women have little choice about how they feed their babies. This was highlighted in the past in this population in 1987-1990 when 18% of infants died. Infantile beri-beri, a form of acute thiamine (Vitamin B1) deficiency in exclusively breastfed infants was recognized and accounted for 40% of all deaths. This has been successfully addressed by prescription of pregnancy and postnatal Vitamin B1 supplements [41].

## Conclusion

Refugees living in Maela camp on the Thai–Myanmar border have a strong breastfeeding culture with high initiation rates and prolonged duration of breastfeeding including amongst premature infants. Women and midwives have expressed certainty and confidence in women’s ability to breastfeed and an acceptance of breastfeeding as an integral part of being a mother. The early initiation of breastfeeding and the close contact between Karen mothers and babies may ameliorate any possible negative effects of swaddling and no SSC on breastfeeding. Maternal nutrition is critical for exclusively breastfed infants. Professional interventions and education should acknowledge and respect cultural and traditional practices that promote, support and protect breastfeeding and maintain a strong breastfeeding culture.

## Abbreviations

EBF: Exclusive breastfeeding; FGD: Focus group discussion; PMTCT: Prevention of mother to child transmission (of HIV); SMRU: Shoklo Malaria Research Unit; SSC: Skin-to-skin care.

## Competing interests

The authors declare they have no competing interests.

## Authors’ contributions

ALW, RM and MMG conceived the study and participated in its design. ALW and M conducted the FGD; ALW conducted the interviews; ALW and RM analysed the data; CPD, MKP, assisted with data collection. RM, ALW interpreted the data and ALW drafted the manuscript. RM, MMG, WS, VIC, FHN revised the manuscript. All authors read and approved the final manuscript.

## Supplementary Material

Additional file 1 Postpartum observation form. Click here for file
