# Supplementary material for: High initiation and long duration of breastfeeding despite absence of early skin-to-skin contact in Karen refugees on the Thai-Myanmar border: a mixed methods study
Source: Int Breastfeed J. 2012 Dec 13;7:19. doi: 10.1186/1746-4358-7-19 (PMC3547777; doi:10.1186/1746-4358-7-19)
Supplement: Additional file 1 — Postpartum observation form. [file 1746-4358-7-19-S1.pdf]

POST PARTUM OBSERVATIONS

NAME____________________________

ANC CODE __ __ __ - __ __ __ __ __ __

EVERY 15 mins for 1 HOUR after birth, THEN at 2,3 and 4 hours

Date of birth __ __ / __ __ / __ __ and time of birth __ __hr: __ __ min

MOTHER												
**Time**		**Fundus**			**Bladder**	**Pass urine**	**Bleeding**	**Temp**	**BP**	**PR**	**RR**	**Action**
		**H or S**	**C or NC**	**fundal height**	**F or NF**	**Y or N**	**S or amount**					
time after delivery	actual time	hard (H) or soft (S)	central (C) or not central (NC)	fingers above (1-3↑) or below (1-3↓) umbilicus	feel (F) or not feel (NF)	yes (Y) or no (N), when Y write amount (in cc, small or large)	if more than slight (S) weight sarong and write amount in cc	° Celsius	mmHg	beats /min	breaths /min	like rub uterus, start PPH-sheet, catheter in-out
**15 mins**												
**30 mins**												
**45 mins**												
**1 hour**												
**2 hours**												
**3 hours**												
**4 hours**												

NEWBORN						
	**Breathing**	**Colour**	**Temp**	**Heart rate**	**Breast feed**	**Action**
time after delivery	breaths / min	pink / blue / white / red	° celsius	beats / min	yes (Y) or no (N), must feed < 1 hour	like give hot water bottle, show to medic, help mother breast feeding
**15 mins**						
**30 mins**						
**45 mins**						
**1 hour**						
**2 hours**						
**3 hours**						
**4 hours**						

First urine: date _ _/_ _/_ _ _ _ time _ _:_ _
First stools: date _ _/_ _/_ _ _ _ time _ _:_ _

Comments:____

Name midwife:__________________________
